# Time dynamics of protein complexes in the AD11 transgenic mouse model for Alzheimer’s disease like pathology

**DOI:** 10.1186/s12868-015-0155-5

**Published:** 2015-04-29

**Authors:** Ivan Arisi, Mara D’Onofrio, Rossella Brandi, Antonino Cattaneo, Paola Bertolazzi, Fabio Cumbo, Giovanni Felici, Concettina Guerra

**Affiliations:** Genomics Facility, European Brain Research Institute (EBRI) Rita Levi-Montalcini, Via del Fosso di Fiorano, 64, 00143 Rome, Italy; Neurotrophic Factors and Neurodegenerative Diseases Unit, EBRI, Rome, Italy; Scuola Normale Superiore, Piazza dei Cavalieri 7, 56126 Pisa, Italy; Istituto di Analisi dei Sistemi ed Informatica “Antonio Ruberti” (IASI-CNR), Rome, Italy; College of Computing, Georgia Institute of Technology, Atlanta, GA USA

**Keywords:** Alzheimers disease, NGF, Neurodegeneration, Protein-protein interaction, Correlation, Network, Protein complex, AD11 mouse model

## Abstract

**Background:**

Many approaches exist to integrate protein-protein interaction data with other sources of information, most notably with gene co-expression data, to obtain information on network dynamics. It is of interest to look at groups of interacting gene products that form a protein complex. We were interested in applying new tools to the characterization of pathogenesis and dynamic events of an Alzheimer’s-like neurodegenerative model, the AD11 mice, expressing an anti-NGF monoclonal antibody. The goal was to quantify the impact of neurodegeneration on protein complexes, by measuring the correlation between gene expression data by different metrics.

**Results:**

Data were extracted from the gene expression profile of AD11 brain, obtained by Agilent microarray, at 1, 3, 6, 15 months of age. For genes coding proteins in complexes, the correlation matrix of pairwise expression was computed. The dynamics between correlation matrices at different time points was evaluated: paired T-test between average correlation levels and a normalized Euclidean distance with *z-score*. We unveiled a differential wiring of interactions in a set of complexes, whose network structure discriminates between transgenic and control mice. Furthermore, we analyzed the dynamics of gene expression values, by looking at changes in gene-to-gene correlation over time and identified those complexes that exhibit a different timedependent behaviour between transgenic and controls. The most significant changes in correlation dynamics are concentrated in the early stage of disease, with higher correlation in AD11 mice compared to controls. Many complexes go through dynamic changes over time, showing the role of the dysfunctional immunoproteasome, as early neurodegenerative disease event. Furthermore, this analysis shows key events in the neurodegeneration process of the AD11 model, by identifying significant differences in co-expression values of other complexes, such as parvulin complex, with a role in protein misfolding and proteostasis, and of complexes involved in transcriptional mechanisms.

**Conclusions:**

We have proposed a novel approach to analyze the network structure of protein complexes, by two different measures to evaluate the dynamics of gene-gene correlation matrices from gene expression profiles. The methodology was able to investigate the re-organization of interactions within protein complexes in the AD11 model of neurodegeneration.

## Background

The importance of understanding biological interaction networks has stimulated the development of numerous techniques, based on different physical principles, for the generation of interaction data and the design of analysis tools for such data. Usually cells networks are represented as graphs whose nodes correspond to molecules (proteins, mRNAs, small molecules that are not encoded by genes, and so on) and edges correspond to various types of relationships among molecules (physical interactions, enzymatic reactions, transcriptional activation, effects of cell signaling components on downstream effectors). As a consequence, many experimental methods have been developed with the aim to generate protein-protein interaction (PPI) networks.

PPI is one of the main organizational principles. PPI networks are complex entities containing tens to thousands of interactions which are rarely annotated with dynamic conditions (such as cellular environment) and location information. Many approaches have been proposed in the literature to obtain dynamic information and integrate PPI data with other sources of information, most notably with gene co-expression values [1-5]. Models of dynamic networks were also introduced in the literature [6,7]. Usually cellular functions are not determined by a single individual gene product: rather they are determined by the interaction of gene products. Thus, it is of interest to look at the behaviour of groups of interacting gene products that form a protein complex, either permanent or transient, or work as a biological process. Proteins complexes have been also investigated from a dynamic perspective [8]. Interesting studies on dynamics of complexes are presented in [9-12].

Computational approaches to identify protein complexes in a network are mainly based on extracting highly interconnected, or dense, subnetworks in a PPI network. Density can be considered in absolute terms, i.e. the number of interactions among proteins in the subnetwork, or can be the ratio of the number of interactions within the complex to the number of interactions with the rest of the network. The computational approaches include MCL [13], CPM [14] and MCODE [15]. Other approaches such as Align Nemo [16] identify conserved complexes in two different species. From a dynamic point of view, complexes can be stable or transient; an example of a stable complex is the proteasome, while an example of transient complex is given by a kinase interacting with its substrate. An interesting study on dynamics of complexes is presented in [9] where it is shown that proteins, that appear to be static in their corresponding gene expression levels during the cell-cycle phases, are recruited by different complexes in different phases. This was discovered by analyzing gene expression data obtained by microarray experiments on the same tissue, in different time points during the cellular cycle and relating these data with the PPI network [10-12]. While the common interpretation of the dynamics of networks and of protein complexes is related to changes over time, it is also of interest to apply the same type of analysis to time series expression data collected under different experimental conditions, for instance healthy/unhealthy individuals, different tissues, or different metabolic conditions.

**Figure 1:**
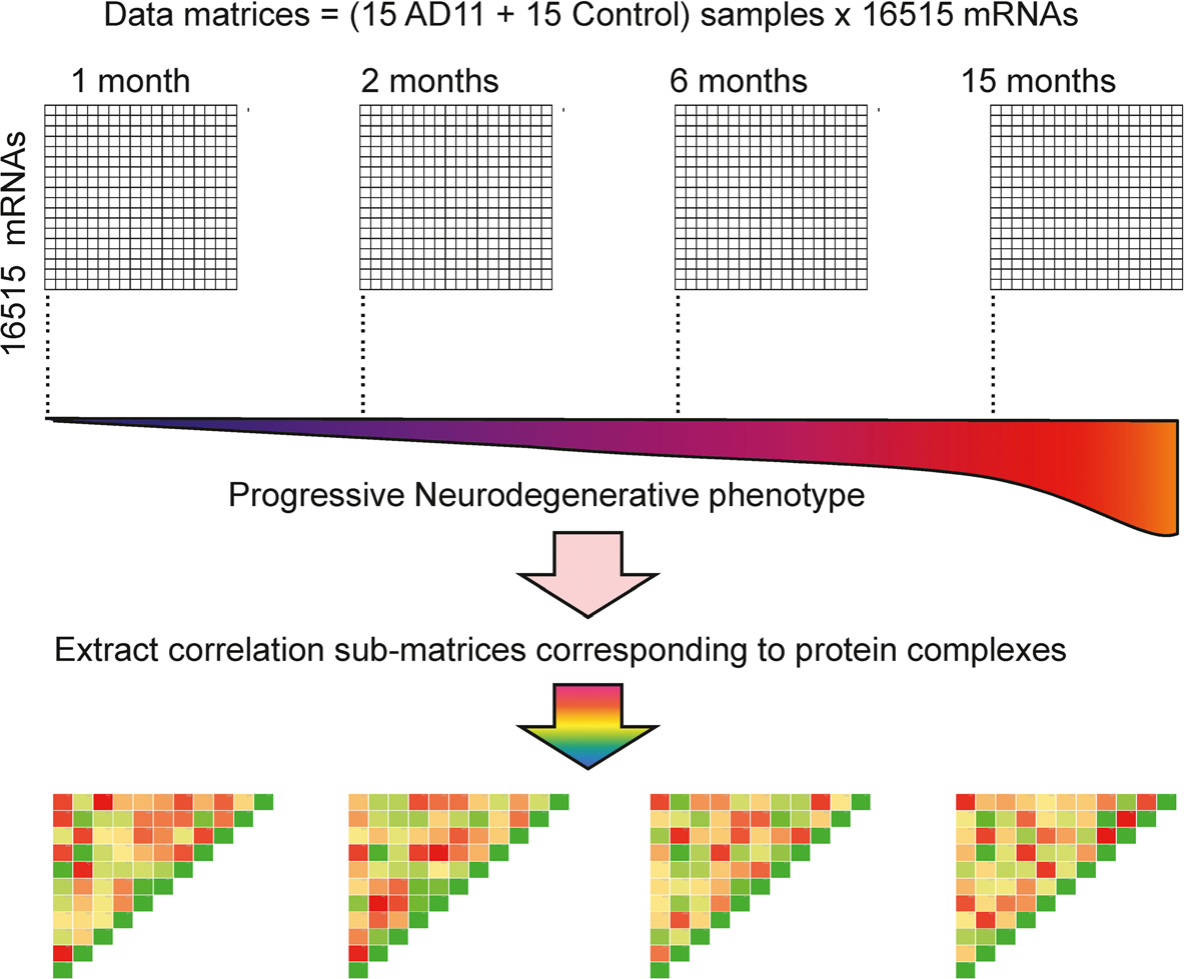
Workflow of analysis. The analyzed dataset includes 16,515 mRNA probes and 30 experiments (15 experiments for controls and 15 experiments for AD11) for each of the four time points (1, 3, 6, 15 months of age), corresponding to the progressive neurodegeneration. From these data, we computed the whole gene-gene correlation matrix and extracted the sub-matrices corresponding to the selected protein complexes, as described in CORUM database (see Methods). Control and AD11 matrices are compared using the chosen metrics.

The main focus of this paper is the analysis of the interaction dynamics of protein complexes in neurodegenerative disease affected mice at different phases of the pathology. We propose an approach to analyze the dynamics of a complex investigating a set of gene expression microarray data [17,18], derived from mice expressing an anti-nerve growth factor (NGF) antibody, called AD11 (see Methods) which develop a progressive neurodegenerative pathology. The AD11 mouse represents a well characterized model of sporadic Alzheimer’s disease [19,20]. The AD11 mice were analyzed at 1, 3, 6 and 15 months of age, versus the corresponding age-matched control mice, with the goal of identifying the early pre-symptomatic pathogenetic events and later markers. The time point of 1 month corresponds to a very early presymptomatic phase of the progressive neurodegeneration. We identified those functional complexes (contained in the analyzed network) that exhibit a different behaviour of constituent proteins, in control and AD11 mice at different stages of the disease. We investigated the difference in the correlation between gene pairs in the known complexes between AD11 and control during the different stages of the disease. Correlation of gene pairs is measured by the value of Pearson Coefficient producing, for each complex, a matrix of gene co-expression values. The workflow of our analysis is outlined in Figure 1. Our approach adopts two different measures to compare the two correlation matrices associated to gene pairs within a complex of AD11 and control: a) one is the difference in the average correlation of the matrices; b) the other is the distance between the two matrices. We compared every protein complex in transgenic and control mice in each time point using the above measures. Data analysis reveals a set of complexes whose dynamics likely discriminate between diseased and control mice. Furthermore, we analyzed the dynamics of gene expression values in AD11 looking at the changes in gene-to-gene correlation over time and identified those complexes that exhibit a different time-dependent behaviour between transgenic and control mice. Concerning the timing of variations along lifespan, the most significant changes in average correlation are concentrated in the early stage of the disease, with a higher correlation in AD11 mice compared to controls. Data analysis shows that many protein complexes are involved in the neurodegeneration and go through dynamic changes over time in AD11 mice.

## Results

### Comparative analysis of gene co-expression values in complexes of AD11 and control over time

We report on the correlation strength of the expression values of genes whose encoded products form protein complexes, and discuss if a different marking is present in such strength between the AD11 transgenic mice and the controls.

We used two different measures to determine the changes of co-expression values:

- the difference of the average co-expression level between AD11 and control matrices;
- the distance between the co-expression matrices of AD11 and control.

The measures (detailed in Methods) differ in one important aspect: while in a) the sign of co-expression values is taken into consideration in the computation of the average, in b) the difference of corresponding elements in the two matrices is taken in absolute value and therefore only its magnitude is taken into account. Therefore the two measures reveal different aspects of the behaviour of the co-expression values.

### Difference of average co-expression levels in complexes

For a given complex of size *n* we computed the co-expression values of the *n(n − 1)/2* pairs of genes encoding the proteins forming the complex using the Pearson correlation coefficient of the gene expression profiles. Such values are arranged in an upper triangular matrix A with *n* rows and *n* columns, where element A_ij_ corresponds to the correlation between the expression values of genes *i* and *j*. This computation was repeated for each of the four time points (months 1, 3, 6, 15), resulting in four matrices denoted by *A*^*1*^*, A*^*3*^*, A*^*6*^, and *A*^*15*^ for AD11 data and by *B*^*1*^*, B*^*3*^*, B*^*6*^, and *B*^*15*^ for the control data. Then, for each of the obtained matrices, the average value was computed over all its entries. At any time point *t,* the difference between the averages of AD11 and control matrices *A*^*t*^ and *B*^*t*^ gives an indication of the correlation change within a complex. To evaluate the significance of the change, we applied the t-student statistical test with a threshold *p* = 0.05 (see Methods). This test generated a binary quadruplet (*t*_*1*_*, t*_*3*_*, t*_*6*_*, t*_*15*_) of +1, 0, −1 numbers, where +1 indicates a significant positive variation AD11 vs. control, −1 a significant negative variation, and 0 no significant modification. The quadruplet associated with a given protein complex enables to immediately visualize at which stage of the disease, if any, a significant difference in the gene-gene correlation of AD11 vs. controls occurs.

Table 1 reports the binary quadruplets associated to complexes of size greater than 3 that are different from (0 0 0 0). It turns out that the complexes are mostly affected at month 1, i.e. at the very early stages of the disease. Precisely, 9 out the 14 complexes listed in the table have a non-zero value as the first element of the quadruplet, with 8 out of 9 values equal to +1, while from 3 months onward only few cases are significant and most of them are equal to -1. This can be interpreted as a general stronger internal correlation in the very early stage of disease in AD11 vs. control mice, while the opposite occurs later in life. This also suggests that most of the crucial events triggering the disease in this model occur very early in life. The majority of analyzed complexes (27 out of 39 with size >3) have difference in average co-expression very close to 0 and are associated with (0, 0, 0, 0) quadruplets (Table 1). They are not reported in the table and will be analyzed from a different perspective in the next subsection.

**Table 1:** **Variation in average correlation levels between AD11 and controls**

		**Quadruplets: AD11 vs. Control**			
**COMPLEX**	**Size**	**t**_**1**_	**t**_**3**_	**t**_**6**_	**t**_**15**_
Parvulin-associated-pre-rRNP-complex	40	**1**	0	0	**−1**
20S-proteasome	14	**1**	0	0	0
immunoproteasome	14	**1**	0	0	0
B-Ksr1-MEK-MAPK-14-3-3-complex	8	0	0	**−1**	0
Gata1-Fog1-MeCP1-complex	8	0	**1**	0	0
Drosha-complex	7	**1**	0	0	0
BLOC-1-biogenesis-of-lysosome-related-organelles	6	0	0	**−1**	0
Metallothionein-3-complex	6	0	0	0	**−1**
Brd4-Rfc-complex	5	**1**	0	0	0
MCM-complex	5	**1**	**−1**	0	0
Agap11-AP3-complex	4	**1**	0	0	0
Kif3-cadherin-catenin-complex	4	**1**	0	0	0
Sarcoglycan-sarcospan-syntrophin-dystrobrevin	4	0	0	**1**	0
Wave-2-complex-Rac-activated	4	**−1**	0	0	0

The binary quadruplet (column 3) represents the significance of the difference in average correlation (obtained from gene expression profiles) between AD11 (disease affected) and control data in a complex at the 4 different time points (1,3,6,15 months of age). Size is the number of genes in the complex. Only complexes with size > 3 and with quadruplet different from (0 0 0 0) are reported. The complexes are listed according to their size. Significant values (+1/-1) are highlighted in bold.

### Distance of co-expression matrices of AD11 and control complexes

To further justify the second type of analysis we observed that the behaviour of the complex might not be the same in AD11 and control, even without significant variation of the average co-expression, for example if the standard deviation of co-expression values within such complexes is too high for a significant *t*-test. Thus we investigated all complexes to check whether we can identify new complexes not revealed by the previous measure. To that end, as mentioned, we used a different measure of similarity of co-expression values, based on the difference of co-expression levels of corresponding pairs of genes in AD11 and controls. In this way we can reveal both positive and negative changes due to single components of the two correlation matrices even if the average of the two matrices is similar.

Given two co-expression matrices, we defined their distance *d* as the square root of the normalized sum of the squared differences over the elements of the matrices (more details in Methods). For each protein complex, such distance is calculated for the four time points, generating a vector representation composed of four positive values $$ {d}_1^{AD11- Control},\ {d}_3^{AD11- Control},{d}_6^{AD11- Control},{d}_{15}^{AD11- Control}, $$$$ \mathrm{e}.\mathrm{g}.\kern0.5em {d}_t^{AD11- Control} $$ is the distance between the two matrices at time *t*. To evaluate the statistical significance of the distance values, we computed the *z-score* of these values by comparing them to control values obtained for random complexes, as explained in Methods section. Table 2 reports the results of this computation applied to AD11 and control data at the four time points. The quadruplets of distance values revealed that for some complexes such values are not uniform; rather, there are peaks indicating a more pronounced variation at some specific time points. Consistently with the results presented in the previous section, we noted that the most significant values of the distance (*z-score* > 1.9) occur at 1 month of age. Indeed, this is confirmed by the average distance $$ {d}_t^{AD11- Control} $$ over all complexes (including those not reported in the table) which is higher at 1 month than at 15 months of age (0.2 and 0.14, respectively). The first 5 complexes listed in Table 2 differ between AD11 and control at 1 month of age, specifically*:* immunoproteasome, Mediator complex, Wave-2-complex-Rac-activated, p97-Ufd1-Npl4-IP3-receptor-complex,Tis7-Sin3-Hdac1- Ncor1-Sap30-complex while for Axin-Dvl-Gsk-Frat1-complex an high value of the *z-score* is present at 3 months of age. The complex Wave-2-complex-Rac-activated is the only one in Table 2 with three high *z-score* values (at month 1, 3 and 15). Furthermore, along with the immunoproteasome complex, Wave-2-complex-Rac-activated was also highlighted in the previous analysis (Table 1). From the results in Table 1 and Table 2, the presymptomatic phase of the disease (1 month) is where the majority of significant changes occur.

**Table 2:** **Distances between correlation matrices of AD11 and controls**

		**Distance: AD11-Control**			
**COMPLEX**	**Size**	**d**_**1**_	**d**_**3**_	**d**_**6**_	**d**_**15**_
immunoproteasome	14	0.26	0.14	0.2	0.16
		**(2.22)**	(−2.45)	(−0.22)	(0.19)
Mediator-complex	7	0.29	0.2	0.16	0.11
		**(2.10)**	(0.09)	(−1.00)	(1.17)
Wave-2-complex-Rac-activated	4	0.33	0.07	0.33	0.36
		**(1.98)**	(−1.85)	**(1.97)**	**(3.39)**
p97-Ufd1-Npl4-IP3-receptor-complex	4	0.34	0.21	0.16	0.20
		**(2.14)**	(0.31)	(−0.56)	(0.86)
Tis7-Sin3-Hdac1-Ncor1-Sap30-complex	4	0.34	0.2	0.12	0.03
		**(2.14)**	(0.16)	(−1.16)	(−1.81)
Axin-Dvl-Gsk-Frat1-complex	4	0.28	0.33	0.13	0.1
		(1.20)	**(2.17)**	(−1.01)	(−0.7)
PYR-complex	10	0.19	0.16	0.17	0.27
		(−0.67)	(−1.22)	(−0.98)	**(3.20)**
PU,1-associated-protein-complex	4	0.21	0.21	0.21	0.3
		0.11)	(0.31)	(0.18)	**(2.44)**
TFIID-complex	7	0.25	0.15	0.17	0.2
		(1.09)	(−1.16)	(−0.76)	**(2.15)**
ORC-complex-origin-recognition-complex	4	0.21	0.18	0.27	0.27
		(0.11)	(−0.14)	(1.07)	**(1.97)**

The distances of AD11 and control matrices at the 4 different time points (1,3,6,15 months of age) are reported in columns (d_1_, d_3_, d_6_, d_15_) respectively. The *z-score* of each value is reported in parentheses below the value itself. Size is the number of genes in the complex. Only complexes with size > 3 and with at least a *z-score* value above 1.9 are reported. Negative *z-scores* indicate a distance AD11 vs. control matrices smaller than the random expectation. Significant *z-scores* are highlighted in bold.

## Discussion

We have investigated, by a correlation analysis, the gene expression data, corresponding to protein complexes, in samples of transgenic mice expressing an anti-NGF antibody and developing a progressive form of neurodegeneration (called AD11), compared to age-matched controls. This led to the identification of complexes showing a relevant difference in co-expression values mostly at 1 month. Some of these complexes will be presented below and the possible functional significance in the neurodegeneration will be discerned.

### Parvulin-associated pre-rRNP complex

**Figure 2:**
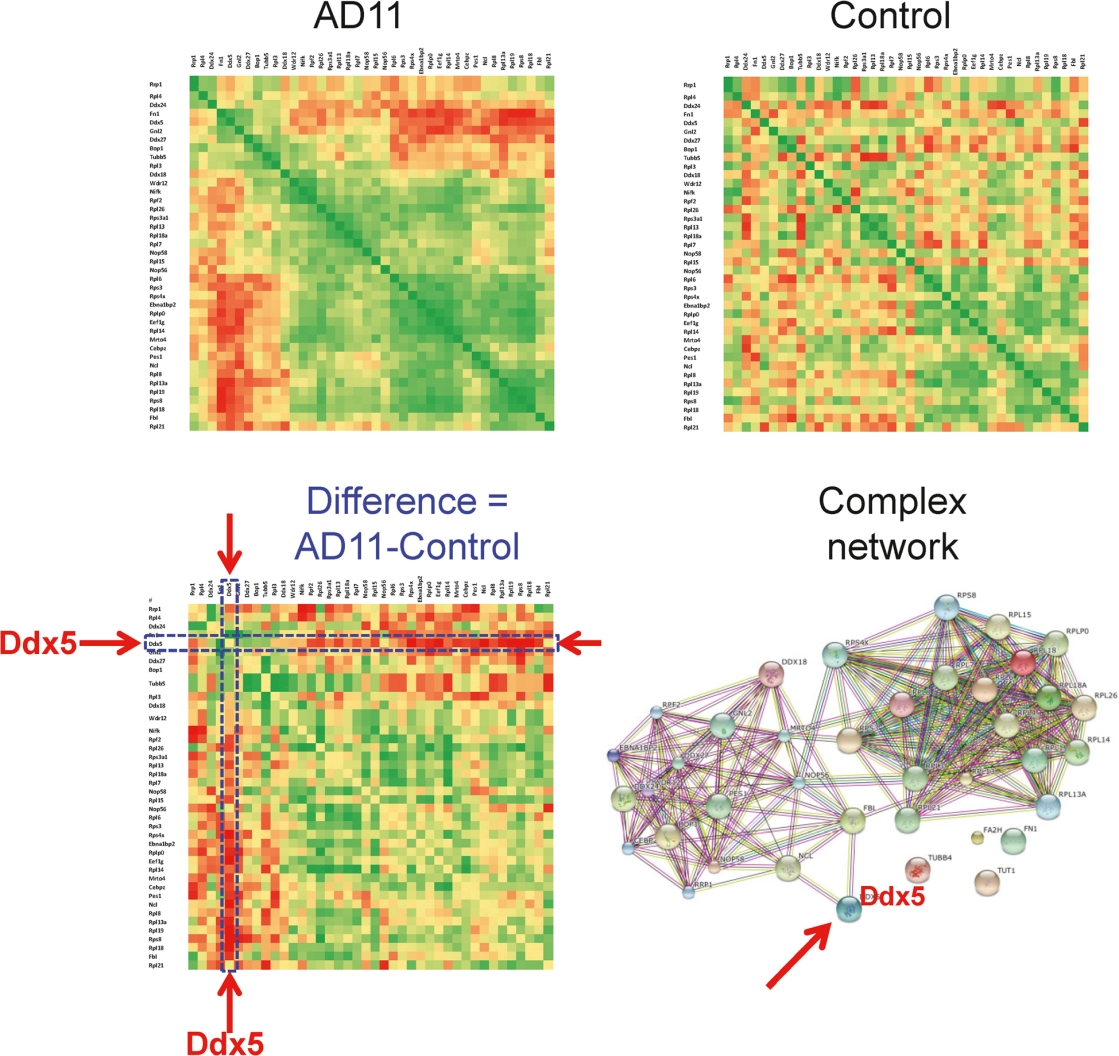
Correlation in Parvulin complex at 1 month of age. Whole correlation matrices of Parvulin-associated pre-rRNP complex in AD11 and control mice (top and center), and the difference matrix (bottom) at 1 month of age. The gene symbols are along the matrix. Correlation values go from −1.0 (red) to +1.0 (green). The correlation structure is globally very different, in particular the red arrows in the difference matrix highlight the Ddx5 gene row and column that show a strong anti-correlation in AD11 compared to control. The protein complex interaction network is shown on the bottom right, as obtained from the StringDB online tool. The Ddx5 protein appears as very poorly connected to the network.

This complex was isolated by immunoprecipitating parvulin from mouse and human cells. The complex is formed by preribosomal RNAs, at least 26 ribosomal proteins and 26 factors involved in rRNA processing and assembly at an early stage of ribosome biogenesis [21]. Those are likely to be involved in ribosome assembly and nucleolar assembly. Human parvulin (hParvulin;Par14/EPVH) [22] belongs to the third family of peptidylprolylcis-trans isomerases that exhibits an enzymatic activity of interconverting the cis-trans conformation of the prolyl peptide bond, and shows sequence similarity to the regulator enzyme for cell cycle transitions, human Pin1. Pin1 is involved in the pathogenesis of certain cancers and protein folding pathologies, in particular aberrant Amyloid processing and Tau hyperphosphorylation like Alzheimer’s and Parkinson’s disease [23-26]. Even though the structure of the complex is significantly different for AD11 and control mice both at 1 month and at 15 months of age, we will focus on the early stage because of our interest in the presymptomatic phase of the disease. Figure 2 shows the whole correlation matrices for the complex at 1 month of age, but also the difference matrix, where the Ddx5 row and column appear as the most anti-correlated within the matrix. Ddx5 (better known as P68) is a member of DEAD box helicases family and a cofactor to a number of proteins involved in brain development and cell proliferation. Ddx5, and its closely associated long non-coding RNA steroid receptor RNA activator (SRA), act in different complexes and signaling systems, including Estrogen Receptor and Notch [27]. Estrogen Receptors, expressed both in neurons and glia, coupled to Ddx5 that shows an age- and sex dependent expression level, are crucial for nervous system development [28]. Ddx5 has also been proved to have a key role in development for Wnt and beta-catenin signaling [29]. The oncogenic role of Ddx5 in cell proliferation and cancer and the role of NF-κB as one of the identified Ddx5 interactors have been proved [30]. In Figure 2 the network structure is shown, as generated by the StringDB online tool: the Ddx5 protein appears as one of the least connected, suggesting this is a critical node of the complex. In our study, the heavy anti-correlation of Ddx5 towards the other complex members, may suggest that at 1 month the complex negatively regulates the active participation of the gene in this specific complex, but not in others, as suggested by the roles in several other cellular functions. Later in life, after 3 months of age in the AD11 model, Ddx5 is recruited again at the same level as control. It has been reported that Ddx5 regulates the splicing of tau gene [31], a protein critical for cytoskeleton maintenance and axonal transport and a key player in AD: this suggests that the early anomaly in the Parvulin complex, may trigger the tauopathy observed that appears in the AD11 model from 2–4 months onwards [18,19].

### 20S Proteasome complex

20S proteasome is a stable complex for which the crystal structure is available in the protein data bank (PDB). Overall data indicate a decline in the Proteasome complex correlation, which is likely to correspond to a decline in the molecular efficiency. Proteasome dysfunction and insufficient removal of misfolded proteins are crucial actors in neurodegenerative pathologies [32-34].

One of the classical hypothesis to explain the pathogenesis of sporadic forms of AD, is a defective clearance of tau and Aβ protein aggregates. The degradation of Aβ depends on a number of different proteases, some of them only active in specific cellular compartments, with the proteasome active in the cytosol [35]. The alteration of this delicate equilibrium may favour intra and extra-cellular Aβ accumulation. Conversely, the Aβ40 and Aβ42 fragments, both in oligomeric and fibrillary forms, were shown to negatively interfere with proteasome complex function *in vitro*, by an inhibition of the enzymatic activity. This suggests a negative feedback further impairing proteasome function [36]. Similarly to Aβ, the abnormal aggregation of misfolded tau protein leads to a neurofibrillary pathology, generating insoluble aggregates common to several neurodegenerative forms, such as AD and other tauopathies. Many experimental findings, though not all of them, show an impaired core-proteasome activity in AD samples [37], suggesting an impairment of the proteasome with ageing followed by an incomplete clearance of tau protein, which is normally soluble and degraded by the proteasome. We can hypothesize that, in AD11 model, a dysfunctional proteasome in the pre-symptomatic phase (1 month of age), should favour an early but progressive accumulation of tau aggregates [38].

### Immunoproteasome complex

**Figure 3:**
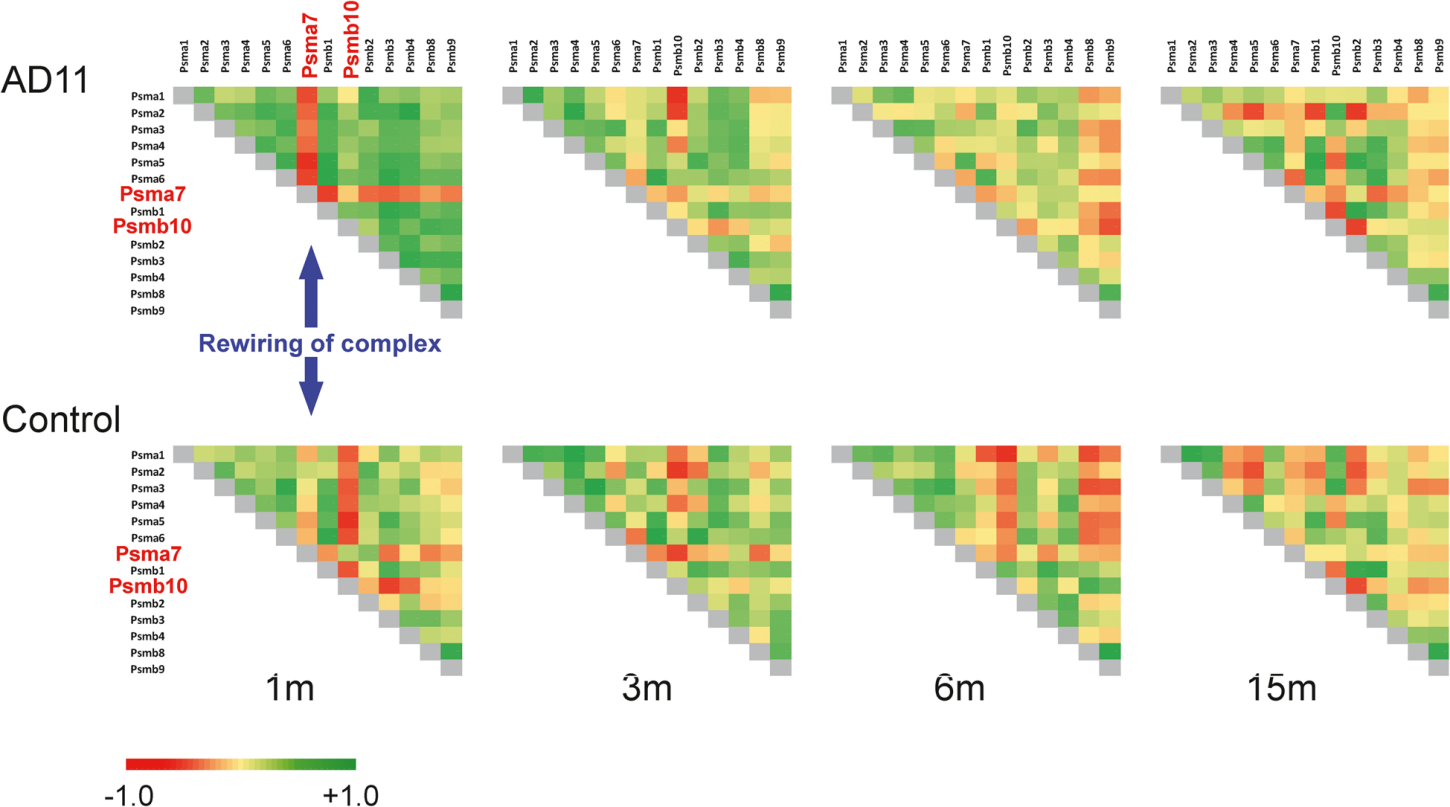
Correlation in immunoproteasome complex. Triangular correlation matrices of immunoproteasome complex in AD11 and control mice at l, 3, 6, and 15 months of age. The gene symbols are along the matrix. Correlation values go from −1.0 (red) to +1.0 (green). The blue arrows indicate that changes in the correlation network are statistically significant only at l month, both from metrics in Table 1 and Table 2. At l month of age, the genes within the complex in AD11 mice are heavily correlated, with the notable exception of Psma7 gene, while in control mice the average correlation is smaller and the only clearly anti-correlated gene is Psmb10. These two genes are highlighted in red.

The immunoproteasome complex is similar to the 20S proteasome, except for the catalytic β subunits Psmb5, Psmb6, Psmb7 that are replaced by Psmb8, Psmb9, Psmb10. Its role is connected to cleaving peptides that will be associated by MHC class I molecules [39], to gamma-interferon induced response [40], but also to protect from oxidative stress, as shown in Psmb10 and Psmb8 (−/−) KO mice [41]. The immunoproteasome complex has been shown to exhibit much reduced stability relative to standard proteasomes, coherently with the capacity of the immune system to efficiently respond to antigens. Moreover, a decreased stability is a mechanism for a rapid but flexible reaction [42]. The immunoproteasome shows different dynamics along lifespan, compared to 20S-Proteasome complex, since the quadruplets in Table 1 are (1,0,0,0) for both, but in Table 2 the 20S-Proteasome complex is absent, highlighting a significantly different network dynamics of these two complexes. At 1 month, the AD11 mouse shows a high correlation between complex members, except for Psma7 gene, which is clearly anti-correlated, thus in some way dissociated from the immunoproteasome network, according to our interpretation of the correlation as measure of protein-protein association. In control mice, a similar anti-correlation occurs with Psmb10 gene, in the context of a general lower correlation between nodes. The general differential correlation between AD11 and control at 1 month disappears at 3 months of age, when both mice show a similar correlation pattern (Figure 3), as if in AD11 the immunoproteasome system takes much longer to reach the physiological state. The difference at 1 month is mainly attributable to: Psma7 anti-correlated to all the other genes in AD11 immunoproteasome complex, Psmb10 anti-correlated mostly to α subunits of complex, and to a lesser extent Psmb9. Psma7 is negatively correlated with interferon based response to viral antigens [43], furthermore Psma7 depletion inhibits cancer growth, being heavily involved in cell cycle and transcription control [44]. The differential behaviour of Psma7 and Psmb10 in our data suggests a dysfunctional immunoproteasome, where a highly correlated Psmab10 in AD11 mice, may have been the consequence of an anomalous immune response or an inflammatory response, able to directly regulate immunoproteasome specific subunits, including Psmb10 [45].

Overall, literature reports that proteasome system is affected in neurodegeneration. Whether the proteasome system, including immunoprotesome, is impaired or over-activated, remains a controversial question, both in human and animal models. Nevertheless, the results seem more in favour of an abnormal activation of immunoproteasome and a decreased activity of proteasome and ubiquitin systems, as shown in human AD and animal models [37,46,47].

Some authors suggest an apparent global down-regulation of the proteasome and immunoproteasome systems [47]. Other findings show a higher activation of immunoproteasome in brain of AD patients compared to the brain of non-demented elderly, being its expression in young brain negligible or absent. Furthermore, AD affected regions show a partial decrease in proteasome trypsin-like activity [37].

This is in agreement with the well known involvement and activation of inflammatory and immune system in human neurodegenerative diseases: a classical example is the activation of astrocytes around Aβ plaques. The different reports may be originated by biological variability and different experimental protocols to assess the proteasome activity, unable to discriminate between the proteasome core units and the immunoproteasome [37].

### Mediator complex

**Figure 4:**
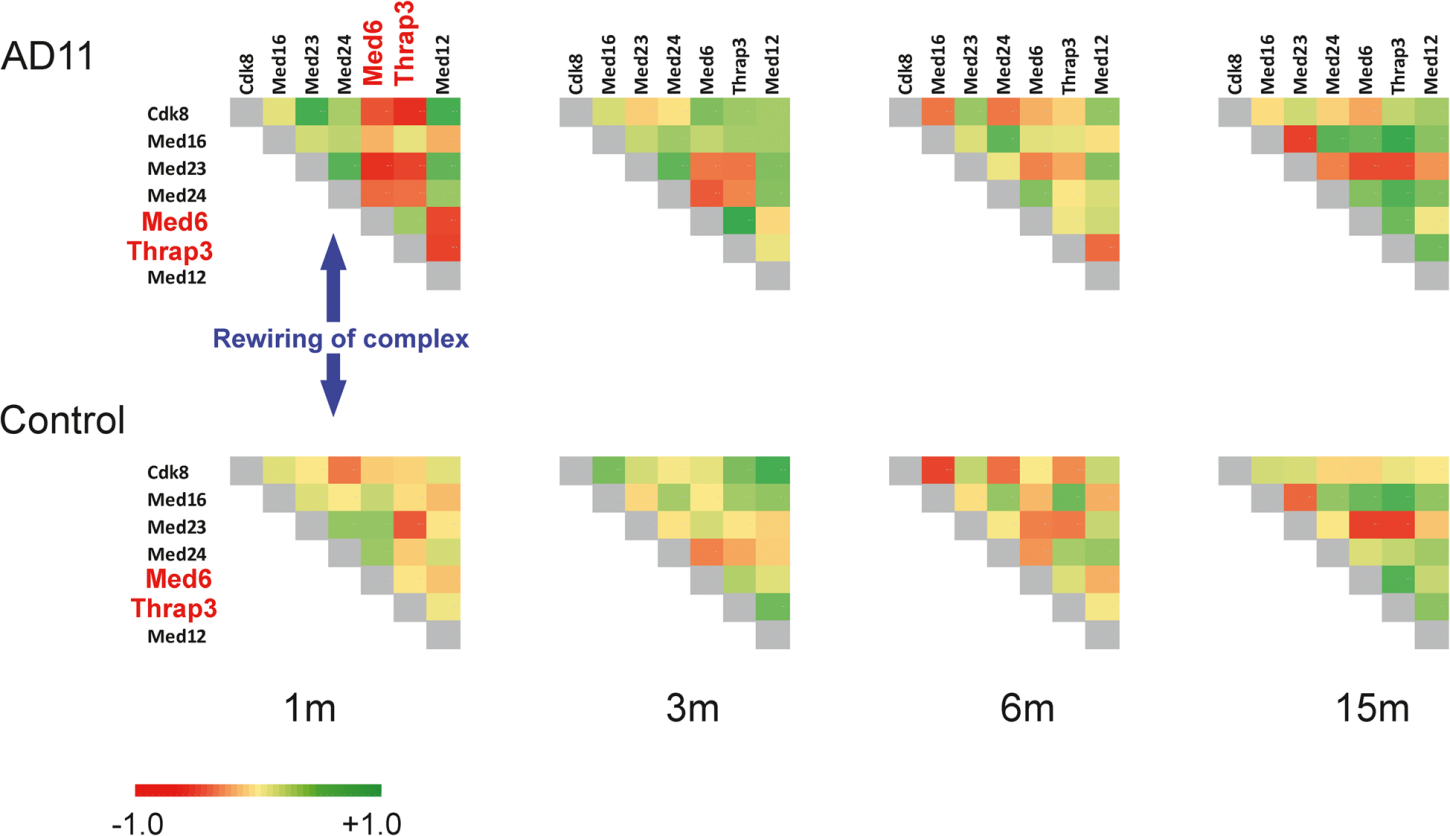
Correlation in Mediator complex. Triangular correlation matrices of mouse Mediator complex (according to CORUM database) in AD11 and control mice at 1, 3, 6, and 15 months of age. The gene symbols are along the matrix. Correlation values go from −1.0 (red) to +1.0 (green). The blue arrows indicate that changes in the correlation network are statistically significant only at 1 month in Table 2. At 3, 6, 15 months of age the correlation matrices are similar, but at 1 month Med6 and Thrap3 (also known as Trap150) are heavily anti-correlated only in AD11 mice. These two genes are highlighted in red.

The mediator complex plays a crucial role in transcriptional mechanisms, acting as a bridge between a large set of transcription factors and RNA Pol II, thus modulating Polymerase activity. This role seems to be mediated by structural modification of the complex [48]. The mediator complex proteins are involved in a large variety of cellular processes such as transcription initiation and elongation, the formation of loops that establish connections between enhancer sequences and promoters, modeling the chromatin structure by mediating its epigenetic modifications [49]. Moreover the role of the complex as a whole has been demonstrated in a variety of syndromes, including malignancies and cardiovascular pathologies [50]. This complex has been shown to regulate also neuronal system development, through the transcriptional modulation of specific genes such as Sox9 [51]. The correlation pattern between AD11 and control mice is essentially similar from 3 months onward, but at 1 month of age Med6 and Thrap3 are anti-correlated in AD11 (Figure 4). This anti-correlation may correspond either to an incomplete complex or to a negative modulation of transcription. Thrap3 closely interacts (as proved by co-immunoprecipitation) with Clock (circadian locomotor output cycles kaput) and Arntl (also known as Bmal1 (aryl hydrocarbon receptor nuclear translocator-like), two genes that form an heterodimer and are key players in the circadian cycle [52] and a number of other fundamental processes including neuronal survival. This underlines the connection between an altered circadian clock and brain ageing [53]. The Med6 subunit is a key component of the head module of Mediator complex [54]. When immunoprecipitated, Med6 seems to mediate the transcriptional modulation through different mechanisms compared to other complex subunits, such as Cdk8; in particular it seems to act indirectly on Polimerase II, without exerting a phosphorylation action [55]. In our data, the anti-correlation of Med6 and Thrap3 in relation to the other subunits may indicate an aberrant general transcriptional control and chromatin maintenance at an early time point of the neurodegenerative process.

### Drosha complex

The Drosha complex is essential for microRNA maturation, a well known subclass of small non-coding RNAs. Along with DBCR8, an RNA-binding protein, it forms the complex known as Microprocessor which processes long primary microRNAs. Drosha functions as the catalytic subunit, while DGCR8 recognizes the RNA substrate. Links between microRNA and neurodegenerative diseases have been well established. The microRNA expression profiles are significantly affected in neurodegeneration, both in the brain of human AD [56] and animal models [57,58] and in peripheral blood, where they are potential disease biomarkers [59,60]. This alterations are not simply downstream disease biomarkers of the pathological state, but the signature of an active role played by microRNAs in the neurodegenerative process, for example interfering with the tau network and amyloid networks in AD [61,62]. In particular, the role of Drosha and Dicer complexes in neurodegeneration has been shown in Huntington’s disease models [63] and Dicer Knock-out models, where Dicer ablation leads to a neurodegenerative phenotype [64]. Therefore, the identification of alterations in the Drosha complex structure, not only underlies a general regulation of gene expression mediated by microRNAs, but appears also critical for the neurodegenerative cascade.

### Metallothionein-3-complex

Metallothionein-3 is a component of a multiprotein complex which very early on was associated with neurological disorders in humans [65]. Numerous successive experiments confirmed this association by showing that MT-3 is decreased considerably (by 30%) in brains of patients with AD [66]. Furthermore ZnMT-3, being able to bind bivalent ions, is neuroprotective against Aβ40 and Aβ42 fragments associated to Cu^2+^*in vitro* and *in vivo* [67,68]. In our study the quadruplet (0 0 0–1) obtained for this complex indicates a significant difference between AD11 and control samples at 15 months of age, and this disfunction in the overt phase of the disease is very similar to what is observed in human AD.

### MCM complex

Minichromosome maintenance (MCM) is a component of the pre-replication complex (pre-RC) and may be involved in the formation of replication forks and in the recruitment of other DNA replication related proteins [69]. MCM proteins are necessary for DNA replication initiation and progression in the cell cycle [69,70]. Members of the MCM family were found to be phosphorylated and associated to neurofibrillary tangles and amyloid plaques in AD neurons [70]. It was also experimentally determined that the interaction of MCM proteins with protein FE65 may contribute to the neuronal cell cycle re-entry observed in Alzheimer’s disease (AD) brains [71]. The fact that in our study MCM complex shows a significant variation in AD11 mice from 1 to 3 months and not afterwards, suggests an early interference with ordinary neuronal cell cycle, which could lead to apoptosis or anomalous proliferation [72].

### Wave-2-complex-Rac-activated

Wave-2 is a member of WASP/WAVE family of the actin cytoskeletal regulatory proteins. Our results indicate a strong change in co-expression values between AD11 and controls using both measures of distance (Table 1 and Table 2). This, in accordance with recent findings reported in the literature, links this protein complex to neurodegenerative pathology: WAVE accumulation was associated to neurofibrillary tangles and Aβ both in the 3xTg-AD animal model and AD human samples [73]. For instance, one component of Wave complex, the protein Nckap1, was found to be markedly reduced in AD-affected human brains [74].

## Conclusions

This work was aimed at analyzing the internal correlations of genes corresponding to protein complexes in the brain of a transgenic model of neurodegeneration, the anti-NGF AD11 mouse. More precisely, we tried to quantify how much the neurodegenerative process affects the protein complexes, by measuring the correlation between gene expression profiles. In our approach, Pearson correlation between gene expression data corresponding to proteins belonging to a certain protein complex is taken as a mediated measure of their physical association, within the experimental conditions. Previous works [6,75-77] have investigated the relationship of protein interactions and gene expression for many of the known protein complexes. These studies have shown significant co-expression, both in terms of similarities of mRNA levels and expression profiles, between the subunits of protein complexes. Building on previous analysis of gene correlation within complexes, we have developed an analysis methodology with the aim of exploiting the behaviour of these correlations in time, i.e., their dynamics in disease vs. control mice. The proposed method is innovative as it considers two different techniques, and their proper statistical testing, to analyze the dynamics expressed by the change of matrices over time. We coupled a more standard evaluation of average correlation to a different metric, in order to detect different dynamical features. This second approach unveiled a hidden dynamics that could not be described by an evaluation of the average connections. Indeed only two complexes appear significant with both approaches (Table 1 and Table 2): the immunoproteasome and the Wave-2-complex-Rac-activated. This suggests that, even if often the average correlation does not change significantly across lifespan, the internal structure undergoes a rewiring of interactions. Concerning the timing of variations along the mouse lifespan, significant changes in average correlation are concentrated at 1 month of age, with a higher correlation in AD11 mice compared to controls. Later in life significant changes occur without a precise timing and are mostly negative, that is in the direction of a smaller average correlation in AD11 mice. A higher correlation at 1 month of age, the first analyzed time point, may suggest that at this early stage the brain complexes show a more rigid structure in the AD11 model compared to control, while at later stages there may be more flexibility, with an opposite behaviour and thus a less correlated network in the AD11 model (Table 1). According to the second used metric, the distance between correlation matrices, statistically significant variations occur both at 1 month and at 15 months of age, that is before the onset and at the end of the disease progression (Table 2). The two different metrics essentially target unrelated features of the complexes, as proved by the fact that many complexes unaffected according to the first metric (not included in Table 1), are significant in the second approach (Table 2). The first metric is a measure of a general strength of the interactions by the average correlation level, while the second metric is a measure of the re-organization rate within the complex: stronger interactions are likely to be associated to less flexible networks and vice versa.

Looking into the detailed structure of the correlation matrix at 1 month of age, we found notable cases of single genes affected within the complexes (Figures 2, 3, and 4), being heavily anti-correlated to most of the other complex members. This is a further level of analysis that targets the connections of single players in the complex: a single node may trigger the full functionality, or a different functionality of the complex, by being connected or un-connected to the network. This is particularly interesting in the case of the immunoproteasome complex, where it is clear that a compact complex, with a correlated general structure in AD11 mice at 1 month of age, is coupled with a strongly anti-correlated Psma7 subunit, while in control mice the un-connected subunit is a different one, Psmb10. The recruitment (transition from uncorrelated to correlated) of different complex subunits at different timing is certainly deeply affecting the biological function. In this respect, we suggest an independent validation of this prediction by targeting protein-protein interactions by monoclonal antibodies, able to disrupt specific nodes or edges inside an interaction network.

We previously characterized transcriptomics profiles in the AD11 model [18] and showed that the absolute peak of differential expression vs. control mice is at 3 months of age in cortex. Interestingly, a significant differential gene expression was identified in the AD11 hippocampus at 1 month of age, mainly related to an alteration in the expression of the inflammation/immune system genes. In the present study the significant changes take place mostly at 1 month and only partially at 15 months. The difference between the present study and our previous one [18] may derive from different mechanisms underlying two distinct phenomena: on the one hand, up- or down-regulation of single genes and, on the other hand, the correlation patterns of functionally related genes that we have associated to protein-protein interactions within complexes. In this study the link of immune system with the neurodegenerative process was confirmed, meanwhile the unexpected role of Parvulin, Mediator and Drosha complexes, highlighted an early involvement and dysregulation of gene expression in neurodegeneration processes, at the transcriptional (Mediator), post transcriptional (Drosha) and translational (Parvulin) levels. Moreover, the identification of proteasome and immunoproteasome complexes highlights the early alteration of protein homeostasis, potentially a prerequisite of misfolding, in the early neurodegeneration process. Since the AD11 mouse at 1 month of age is pre-symptomatic, because there is no apparent neurodegenerative and/or behavioural altered phenotype, this finding is even more significant. A dysfunction in the interaction structure of key critical protein complexes parallels a gene expression alteration.

We plan to validate our results by the use of a proteomic methodology, thus a global approach such as mass spectrometry or protein arrays, coupled to the isolation of single complexes, since this is now feasible by recent techniques [78]. A proteomic approach will allow, for example, to assess if the correlation/uncorrelation status of specific proteins is associated to a stable/transient membership to corresponding complexes, and to find a relation between genomic and proteomic profiles.

We conclude that this novel approach is able to unveil an internal rewiring of interactions within protein complexes, even when there is no apparent change in the average correlation level. The neurodegenerative process in the AD11 mouse model is affecting not only the global gene expression profile along lifespan, but also the efficiency of key molecular machinery, by modulating the interaction network within crucial protein complexes. These might be key events in the pathogenesis and development of AD.

## Methods

Our analysis integrated gene expression data with PPI data. Time series gene expression data correspond to two different classes: AD11 and control mice. PPI data were derived from public datasets. In the following we provide details.

### Microarray dataset

AD11 transgenic mice [19,20] express a recombinant version of the monoclonal antibody mAb αD11 that specifically recognizes and neutralizes NGF [79,80]. AD11 anti-NFG mice were produced by crossing mice that express the heavy chain of the transgenic antibody (VH mice) with mice that express the light chain of the antibody (VK mice). As in previous studies [18], VH mice were used as transgenic control mice that are consistently negative with respect to all the classical neurodegeneration markers (molecular, cellular and behavioural). The brain tissue from a total of 60 female AD11 mice and 60 female VH control mice was used for this study, divided into 15 AD11 and 15 controls for each of the four time points (1, 3, 6, 15 months of age).

The microarray data for the transgenic mouse were provided by the European Brain Research Institute (EBRI, Roma, Italy). The gene expression dataset was obtained by Agilent two-color microarray platform (44 K and 4x44K mouse whole genome chips, grid ID 012694 and 014868). The whole dataset is publicly available from the Gene Expression Omnibus database.

The dataset includes 16,515 mRNA probes and 30 experiments for each time point (15 experiments for controls and 15 experiments for AD11), for a total of 120 experiments. The output gene profiles for the entire genome is thus represented by 8 matrices with 15 columns and 16,515 rows, each row representing one mRNA expression profile. Filtering was applied to the microarray data (Figure 1). Since different probes may match the same gene, we selected only the probes that allow the best discrimination between control and AD11 classes. To do that, we used the ANOVA (Analysis of Variance) test, a statistical technique to analyze the variation in a response variable (continuous random variable) measured under conditions defined by discrete factors. After this initial filtering, we also removed the probes corresponding to genes not present in the protein complexes considered in this analysis, as described in CORUM database (see below). The final dataset contains 559 mRNA probes.

### Protein complexes

Protein complexes annotation was extracted from the Comprehensive Resource of Mammalian protein complexes database (CORUM) [81]. This database provides a resource of manually annotated protein complexes from mammalian organisms. Annotation includes protein complex function, localization, subunit composition, literature references and more. All information included in CORUM was obtained from individual experiments published in scientific articles (unreliable data from high-throughput experiments are excluded). Of the CORUM dataset we considered the subset of protein complexes for which the gene expression data were available in our dataset. Furthermore, after this filtering that reduced the size of a number of complexes we selected the complexes with size greater than or equal to 3, resulting in a final set of 81 complexes of size ranging from 3 to 40.

### Constructing the gene co-expression matrices associated to complexes

The most widely used choice for quantifying gene co-expression are covariance-based measures like Pearson correlation (linear measure) or entropy-based like the mutual information (nonlinear measure). These simple pairwise similarity measures are computationally tractable, although they may suffer from high false discovery rate, i.e., genes are erroneously associated while in truth they only indirectly interact through one or more other genes. We used Pearson correlation of gene expression profiles to infer the co-expression of a pair of genes within the same complex. In this way we computed *n × (n − 1)/2* pairs of values for a complex with *n* proteins and represented them using an upper triangular matrix indexed by the proteins IDs.

### Gene correlation strength within protein complexes

**Figure 5:**
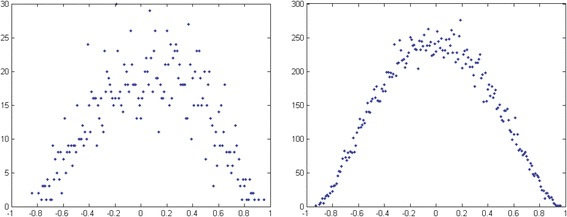
Distribution of correlation values. The distribution of correlations of gene expression profiles within protein complexes in the control mouse at 1 month of age. On the x-axis there are the co-expression values of pairs of proteins within complexes. The complexes are separated into two classes, based on the number N of genes: small with N < =10 (left) and large with N > 10 (right).

As a preliminary assessment, the question of whether there exists a relation between the similarity in gene co-expression profiles and protein complexes was addressed in our transgenic control mice vs. wild type. As a first step, we checked if in the chosen control mice there was a bias of the overall gene-gene correlation structure of expression profiles: at this purpose we first compared the control mouse profiles to publicly available wild mice profiles. We computed the Pearson correlation for each pair of genes in the same complex using the dataset of control mice at 1 month of age. As mentioned, we used CORUM [81] as the reference dataset for protein complexes. The number of genes in complexes of our dataset varies considerably, ranging from 3 to 40, but the vast majority of the complexes are small. For instance, around 95% of protein complexes have size smaller than 10 genes. Thus, as in previous works, we divided complexes into two classes: small (<=10) and large complexes (>10) and analyze them separately [76]. The distributions of the correlations in small and large complexes are shown in Figure 5 for the control mouse at 1 month of age.

Both distributions appear to be centered around zero. Thus, unlike previous studies conducted on yeast and human cells [6,75-77], we did not observe a shift to the right (average positive correlation) of the distribution of large complexes of control mouse. To verify whether this was a property peculiar to the control mouse strain chosen in this study, we performed the same preliminary analysis also using the wild type mouse dataset E-GEOD-4734 available from the public gene expression repository Array Express [82], which contains five mouse brain regions including hippocampus in six mouse strains. The distributions for the wild type are very similar to the ones from the VH control mouse with average value close to zero. Therefore we concluded that the chosen control was suitable as reference for the analysis carried out in this work.

### Variation in average gene co-expression

For a given protein complex, let A and B be the matrices of co-expression values associated with genes inside the complex of AD11 and control samples, respectively, at the same time point. For each matrix, the average was taken over all the elements of the upper triangular matrix (excluding the diagonal). The difference of the averages of A and B was then computed and this computation was repeated for all four time points. This statistic gives an idea of the variation over time of the expression levels of genes inside a complex for the two classes of samples.

A statistical test to evaluate the significance of the change in average co-expression value within a complex was applied. A natural correspondence between data values at the same positions in the two matrices, i.e. *A*_ij_ and *B*_ij_ corresponding to the same two genes *i* and *j*, suggested the use of a paired *t*-student test. Precisely, the paired *t*-student test computes the value:

$$ t=\frac{{\displaystyle {\sum}_{i,j}}\left({A}_{ij}-{B}_{ij}\right)}{\sqrt{\frac{N{\displaystyle {\sum}_{i,j}}{\left({A}_{ij}-{B}_{ij}\right)}^2-{\left({\displaystyle {\sum}_{i,j}}\left({A}_{ij}-{B}_{ij}\right)\right)}^2}{N-1}}} $$

Where *N = n × (n-1)/2* and *n* is the size of a complex. If *t* exceeds the *t* distribution reference value for a certain probability value, then the averages are significantly different at that probability level. We chose, as is generally the case, the probability value *p* = 0.05 (*p-value*).

The significance variations in the averages at the 4 time points (1, 3, 6, 15 months) is represented by: ${t}_1^{AD11- Control},\ {t}_3^{AD11- Control},{t}_6^{AD11- Control},{t}_{15}^{AD11- Control}$.

### Distance of gene co-expression matrices

Given two correlation matrices *A* and *B* corresponding to AD11 and control samples at the same month, we adopted the Euclidean distance between the two matrices as an inverse measure of their similarity. It is defined as the square root of the average squared difference between the elements of the matrices. The formula below represents such measure:

$$ d=\sqrt{\frac{{\displaystyle {\sum}_{i=1,N-1;j=i+1,N}}\ {\left({A}_{ij}-{B}_{ij}\right)}^2}{N}} $$

where *N = n × (n − 1)/2* and *n* is the size of a complex. We note that the proposed measure weights the correlation between all pairs of genes in the same way, and does not possess a theoretical maximum value (its minimum being 0). This does not allow any normalization of *d*. Thus we resorted to a statistical test to provide a significance measure of *d*. We determined the average distance of AD11 and control for a collection of random complexes, then computed the *z-score* for the distance value *d* of an observed complex with respect to the random complexes. Precisely, for each complex size *n* we generated a set of 1000 random complexes consisting of *n* randomly selected genes and their expression values. We did that for each of the two classes AD11 and control and for each of the four time points. The significance *z* of the observed *d* at a given time point and for any given complex size n was transformed into a *z-score* through the relation z = (d − < x >)2/*S*, where *< x >* is the average over all random samples of size *n* of the distances between AD11 and control matrices, and *S* denotes the standard deviation of the same distances. This *z-score* represents the chance that a random complex, consisting of a group of *n* randomly selected genes, could exhibit a distance of gene co-expression matrices between AD11 and control greater than or equal to that of the observed complex. In our test we chose a *z-score* of 1.9 as a threshold of significance.

### Availability of supporting data

The whole Agilent microarray dataset used in this study is publicly available from the Gene Expression Omnibus database (Series ID GSE63617), at the following URL: http://www.ncbi.nlm.nih.gov/geo/query/acc.cgi?acc=GSE63617.
